# Experimental Study of the Application of Calcined Shield Muck Powder as a Substitute for Fly Ash in Synchronous Tunnel Grouting Materials

**DOI:** 10.3390/ma18030482

**Published:** 2025-01-21

**Authors:** Wei Liu, Enfeng Wu, Hangyu Du, Hu Liu, Suyun Liu, Kangqi Chang, Yongqiang Li

**Affiliations:** 1 College of Civil and Transportation Engineering, Shenzhen University, Shenzhen 518060, China; liuwei@szu.edu.cn (W.L.); 2200471074@email.szu.edu.cn (E.W.); 2100471021@email.szu.edu.cn (H.D.); 2 Institute of Technology for Future Industry, Shenzhen Institute of Information Technology, Shenzhen 518172, China; 2021000285@sziit.edu.cn (H.L.); 2021000317@sziit.edu.cn (S.L.); 2024000219@sziit.edu.cn (K.C.)

**Keywords:** shield mud, supplementary cementitious material, pozzolanic reactivity, synchronous grouting material, microscopic performance

## Abstract

During shield tunnel construction, waste mud is a significant source of urban construction waste. However, the disposal of waste mud has always been a challenge in engineering. Addressing the challenge of harmlessly disposing of, or repurposing, mud cakes formed after pressure filtration of shield mud remains a pressing issue for many cities. To address the challenge of shield mud disposal and explore the utilization technology of this resource, this study focuses on shield mud obtained from the Shenzhen subway tunnel. Calcined shield mud powder (CSMP) was prepared by activating its potential pozzolanic properties through a calcination process. Compressive strength tests revealed that, while CSMP exhibits some pozzolanic activity, its performance is limited. When 30% of the cement is replaced, the mortar’s maximum strength activity index (SAI) is only 82.6%, which makes it unsuitable as a supplementary cementitious material for concrete applications. At the same time, CSMP was also evaluated as a partial replacement for fly ash in the formulation of synchronous grouting materials, with performance metrics including fluidity, bleeding rate, hardening rate, setting time, and compressive strength systematically tested. The experimental results showed that, while CSMP reduces the fluidity of grouting, it significantly improves volumetric stability, shortens setting time, and enhances mechanical performance. Compared to the fly ash used in the study, CSMP exhibited better pozzolanic reactivity, promoting the formation of C-S-H and C-A-S-H phases, optimizing the pore structure, and increasing the density and overall performance of the grouting material. When the substitution rate is below 60%, the performance of grouting meets standard requirements, indicating the strong feasibility of utilizing CSMP to replace fly ash in synchronous grouting materials.

## 1. Introduction

The shield tunneling technique is a method of constructing tunnels by using a protective shield to support the ground while excavating and installing tunnel lining segments behind it. The shield tunneling method is a widely adopted construction technique in urban metro and rail transit projects. This method is favored for its high construction efficiency, advanced automation, and provision of a safe working environment [1,2]. Meanwhile, the volume of shield mud generated during shield tunnel construction continues to increase annually, making it a significant contributor to urban construction waste [3,4]. Assuming a typical tunnel diameter of 6 m and a loosening coefficient of 1.5, approximately 45,000 m^3^ of shield mud is generated per kilometer of subway tunnel construction. By 2023, a cumulative production of over 400 million m^3^ of shield mud have been produced [5]. However, shield mud is primarily handled through landfill or stockpiling methods, which consume vast land resources and lead to considerable material wastage [3,6]. Additionally, the widespread use of additives, such as foam agents, bentonite, and chemical modifiers, during shield tunneling, results in discarded slurry with high water content and poor mechanical properties. The large-scale accumulation of shield mud poses significant environmental risks, including landslides and groundwater contamination [7,8]. Therefore, the effective and sustainable disposal and resource utilization of shield mud are pressing challenges that must be addressed to support sustainable urban development.

Multistage mud-sand separation is a promising resource utilization technology for managing shield mud [9,10]. This process involves extracting and cleaning the gravel and sand components from the shield mud, enabling their reuse in construction applications. The residual mud tailwater is then subjected to flocculation and filtration processes for volume reduction, effectively minimizing waste and environmental impacts [11]. The separated and cleaned gravel and sand can be directly recycled as coarse and fine aggregates for concrete [12,13]. However, the resource utilization pathways for the filter cakes formed from the tailwater mud after dewatering remain significantly limited. At present, the main approaches for processing shield mud filter cakes into building materials include their use as raw materials in the manufacture of non-fired bricks and geopolymers [14,15,16]. However, the actual utilization rate of shield mud filter cakes is less than 1% in the above applications [3]. Shield mud is mainly composed of clay minerals such as illite, kaolinite, and muscovite, as well as natural minerals like quartz, calcite and feldspar [17]. When subjected to appropriate thermal treatment, clay minerals like kaolinite undergo dehydroxylation to form metakaolin, enhancing the pozzolanic reactivity of the clay minerals and giving them the functional properties of pozzolanic materials with potential applications as construction materials [18,19]. Hao et al. [20] investigated the feasibility of utilizing shield mud as a cementitious material. Their study revealed that the strength activity index of calcined shield mud generally meets the standards for Class II fly ash. The pozzolanic reactivity of calcined shield mud is closely associated with its kaolinite content. However, compared to pure kaolinite, shield mud derived from engineering waste typically contains lower kaolinite levels, leading to moderate reactivity after calcination. As a result, its performance as a concrete admixture is limited in both effectiveness and dosage. Similarly, Zheng et al. [21] observed that incorporating calcined engineering waste into concrete at levels exceeding 10% adversely affects its mechanical properties and significantly diminishes the workability of the concrete mixture.

Synchronous grouting materials are commonly employed to fill the gaps between shield tunnel segments and the surrounding strata. In the production of synchronous grouting, cement is used as the primary binder, which leads to significant energy consumption and the emission of harmful gases [22]. As a result, the incorporation of binders such as fly ash and bentonite has become a trend [15,23]. Due to the relatively low strength requirements of hardened synchronous grouting materials, the reactivity demands for cementitious components are not particularly stringent. Additionally, workability properties, such as fluidity and consistency, are relatively easy to satisfy. Related studies have also shown that active clay can simultaneously improve the pore structure of materials and reduce penetration and permeability, thereby enhancing the long-term durability of the materials [18,24,25]. Consequently, replacing a portion of cement with ground and calcined shield mud powder, or using it as a supplementary cementitious material in place of fly ash, presents a feasible approach.

To explore diverse pathways for the resourceful utilization of shield mud in construction materials, this study focuses on shield excavation waste from the Shenzhen region. The pozzolanic activity of calcined shield mud powder (CSMP) was evaluated through compressive strength tests. Additionally, ground and calcined shield mud powder was used to replace fly ash as a supplementary cementitious material for preparing synchronous grouting materials. The feasibility of applying CSMP in synchronous grouting materials was assessed by testing workability, bleeding rate, hardening rate, and compressive strength. Furthermore, the microstructure and hydration products of the samples were analyzed using scanning X-ray diffraction (XRD), electron microscopy (SEM), and mercury intrusion porosimetry (MIP) to determine the reaction mechanism of CSMP in synchronous grouting materials.

## 2. Materials and Methods

### 2.1. Materials

**Figure 1:**
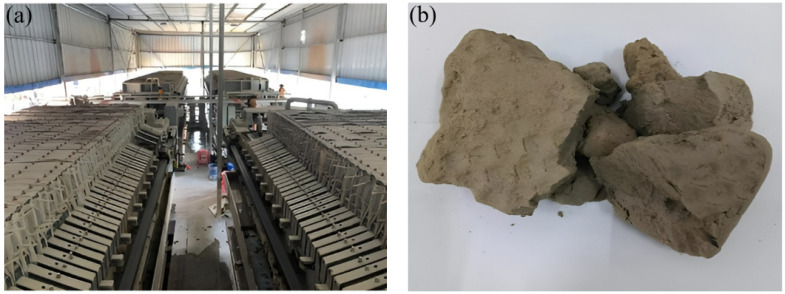
(**a**) Shield mud filtering production line; and (**b**) filtered muck cake.

The basic properties of Conch P.O 42.5 cement produced by Conch Group Co., in Shenzhen, China, are shown in Table 1, and its chemical composition is shown in Table 2. The shield mud was taken from the shield tunnel section of the Ping Shang area on Shenzhen Metro Line 14. First, the slurry was subjected to mud-sand separation followed by filtration, to obtain the mud cake [26]. The production line for shield slurry filtration at the construction site and the dewatered mud cake are shown in Figure 1. The main chemical composition of the shield mud cake is shown in Table 2. The Class II fly ash was obtained from the Mawan Power Plant in Shenzhen, China and its chemical composition is given in Table 2. Commercial sodium-based bentonite produced by Xingda Industry and Trade Co., in Sichuan Province, China, was used, which appears as a white powder with an expansion rate of 16 mL/2g; its chemical composition is shown in Table 2. For sand, China Xiamen ISO standard sand was utilized.

### 2.2. Characterization Methods

#### 2.2.1. Calcination and Reactivity Analysis of Shield Mud

**Figure 2:**
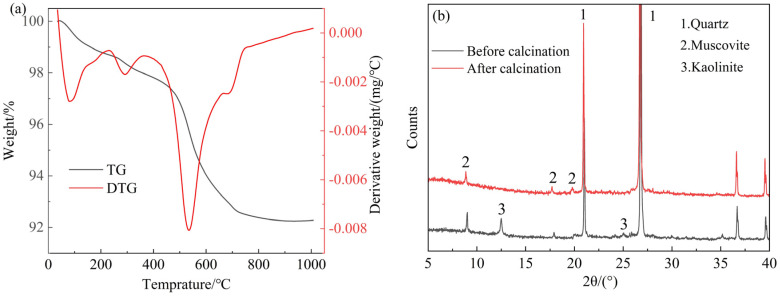
(**a**) Thermogravimetric analysis of SMP; and (**b**) XRD patterns.

The shield mud was dried at 105 °C until a constant weight was achieved, then ground to obtain shield mud powder (SMP). Figure 2 presents the thermal analysis (DTA) and differential X-ray diffraction (XRD) patterns of the SMP before and after calcination. Figure 2a shows that the mass loss of SMP at 250 °C is likely due to the evaporation of moisture adsorbed on the surface and interlayer regions of the clay minerals. This suggests that clay minerals such as muscovite, illite and bentonite may be present in the shield mud. The mass loss (0.6%) observed in the range of 250–350 °C corresponds to the decomposition of iron oxides. The dehydroxylation of clay minerals occurs between 350 and 900 °C. The thermogravimetric (DTG) curve shows a significant mass loss (3.7%) between 400 and 600 °C, which is attributed to the dehydroxylation of kaolinite into metakaolin [27,28]. Between 600 and 900 °C, the curve gradually stabilized, with minimal mass loss (1.8%), indicating that the dehydroxylation of kaolinite was completed within this temperature range. To ensure the full transformation of kaolinite into metakaolin, the optimal calcination temperature should range from 700~800 °C, with a duration of 2–3 h [29]. As presented in Figure 2b, after calcination, the kaolinite peaks in the X-ray diffraction (XRD) spectrum between approximately 10 and 15° nearly disappeared, confirming that kaolinite had been completely dehydroxylated and transformed into metakaolin.

**Figure 3:**
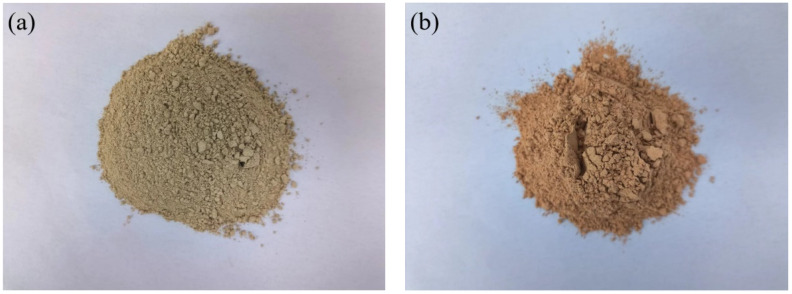
Appearance of shield muck and shield muck powder after grinding and calcination. (**a**) After grinding; (**b**) After calcination.

**Figure 4:**
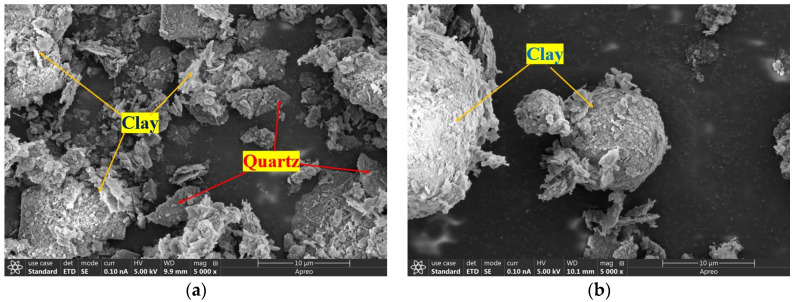
Morphology of shield muck powder before and after calcination. (**a**) Before calcination; (**b**) After calcination.

After calcination, the SMP remained in a powdered form, with its color changing slightly from gray to pale yellow, as shown in Figure 3. The scanning electron microscopy (SEM) images of the powder before and after calcination are shown in Figure 4. As observed in Figure 4, the pristine clay minerals exhibit an irregular pseudo-laminar structure, while the quartz particles display good crystallinity with irregular shapes, flat surfaces, and sharp edges. After high-temperature calcination, the structure of the clay particles was disrupted, leading to agglomeration and the formation of dense spherical structures. This suggests that high calcination temperatures cause the agglomeration and sintering of clay particles [21,30].

The kaolinite component in the SMP was converted into amorphous metakaolin after high-temperature calcination, endowing the calcined shield mud powder (CSMP) with pozzolanic activity. The extent of this activity depends on the kaolinite content and can be quantified using the strength activity index (SAI), which is derived from the compressive strength of mortar specimens. The compressive strength tests were conducted according to the specifications outlined in GB/T 17671-2021 [31]. In the experiments, cement was partially replaced with CSMP at substitution ratios of 0, 10, 20, and 30% by weight, with a consistent water-to-binder ratio of 0.5.

The pozzolanic activity of CSMP was evaluated using both the SAI and the relative strength activity index (RSI) methods. The SAI (%) is an indirect method for evaluating the activity of pozzolanic materials. According to the ASTM C618 standard [32], the formula for calculating the SAI is as follows:

(1) $$\mathrm{SAI}=\frac{A}{B}\times 100\%$$

where A and B are the compressive strength of a mortar sample (with CSMP) and a control sample (without CSMP) at selected curing times (MPa), respectively. For pozzolanic materials used in engineering, their SAI (%) after 28 days should be greater than 75%.

RSI is another method used to evaluate the pozzolanic reactivity of materials, taking into account the influence of the pozzolanic material content. When the material does not exhibit pozzolanic reactivity or shows a positive effect, the strength loss of the material should be proportional to the amount of pozzolanic material added. The RSI provides a more direct reflection of the effect of pozzolanic materials on cement-based materials. The calculation process is as follows [30]:

(2) RSI=RP−(100%−S)

where RP is the ratio of the compressive strength of the test sample to that of the control sample (with the same value as SAI), and S is the percentage of cement replacement (%).

#### 2.2.2. Performance Testing of Synchronous Grouting Materials

In engineering, the performance of synchronous grouting materials is typically evaluated using parameters such as fluidity, consistency, bleeding rate, hardening rate, setting time, and unconfined compressive strength. Fluidity and consistency are two critical indicators of the pumpability of synchronous grouting materials [33].

The bleeding rate and hardening rate reflect the stability of synchronous grouting materials and the filling performance after material hardening. A lower bleeding rate indicates better material stability; whereas, a higher rate indicates poorer stability, increasing the risk of pipeline blockages during the grouting process [34]. In engineering practice, it is essential to ensure that synchronous grouting materials have a high hardening rate, to minimize excessive volume shrinkage, which could otherwise result in ground loss.

Setting time is an essential factor for evaluating the performance of synchronous grouting materials. The grouting material will quickly lose its fluidity, potentially causing difficulties in pumping, clogging of the pipeline, and inability to fill all voids, If the setting time is too short [35]. On the other hand, if the tunnel lining exits the shield tail before the synchronous grouting material develops sufficient early strength, due to an excessively long setting time, the lining remains in a liquid medium and is susceptible to uplift from buoyancy forces [32]. Therefore, both the setting time and compressive strength of synchronous grouting materials play a critical role in ensuring smooth grouting operations and effectively controlling tunnel lining uplift during construction.

The mix proportions for synchronous tunnel grouting materials are typically determined by the following ratios: F/A (ratio of fly ash to cement by mass), B/S (ratio of binder to sand by mass), W/B (ratio of water-to-binder by mass), and P/W (ratio of bentonite to water by mass). For this study, the baseline mix proportion of synchronous grouting material was selected as follows: F/A = 1.9, B/S = 0.58, W/B = 0.87, and P/W = 0.1. The ratios of CSMP replacing fly ash in the synchronous grouting material are 0%, 20%, 40%, 60%, 80%, and 100%, respectively. The properties of the reference synchronous grouting material used in the tests are shown in Table 3, which meet requirements specified in T/CECS 563-2018 [36].

**Figure 5:**
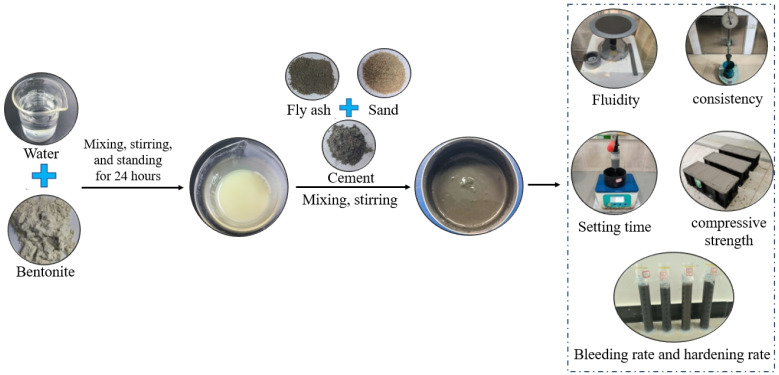
Preparation process of synchronous grouting materials.

In order to prepare the synchronous grouting material, bentonite and water were first mixed and allowed to rest for 24 h. Then, cement, fly ash, CSMP, and sand were combined with the mixture. A uniform grout mixture was obtained after thorough mixing. Once the grouting material was mixed, the initial workability was evaluated by testing its fluidity and consistency. Part of the grouting material was used to test its bleeding rate, hardening rate, and setting time, while the remaining sample was grouted into molds and cured under controlled conditions to assess its unconfined compressive strength at various curing ages. The preparation and performance testing process of grouting is shown in Figure 5.

The properties of synchronous grouting materials were characterized by the parameters of consistency, setting time, and compressive strength, according to JGJ/T 70-2009 [37]. Fluidity was measured using the truncated cone method in GB/T 2419-2005 [38]. Bleeding rate and hardening rate were referenced from T/CECS 563-2018 [36].

#### 2.2.3. Experimental Methods for Testing Microstructure

For scanning electron microscopy, the central portions of the compressed specimens were employed after treating with hydration termination. The process of hydration termination involved crushing the samples and immersing them in isopropanol for 15 min to remove the moisture from them. The crushed samples were then soaked in ether solution for approximately 1 min, enabling secondary replacement to remove the residual isopropanol, followed by vacuum-drying the samples in an oven at 40 °C [39]. A Zeiss Gemini scanning electron microscope was used to observe the microscopic morphology of the samples. After water removal, the samples were milled (through 200 mesh sieve) for XRD testing. The test was conducted using a Bruker D8 Advance X-ray diffractometer (Bruker, Karlsruhe, Germany) with a testing voltage of 40 kV, current of 40 mA, scanning range of 5–70°, and scanning speed of 0.04°/s. The mercury intrusion porosimetry (MIP) test was conducted using the MicroActive AutoPore V 9600 instrument, with a pore size testing range of 5 to 10,000 nm and a pressure range of 30,000 psi.

## 3. Results and Discussion

### 3.1. Reactivity Analysis of CSMP

**Figure 6:**
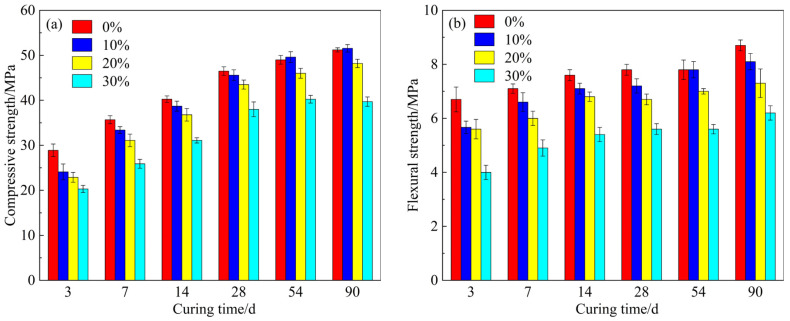
(**a**) Compressive strength; and (**b**) Flexural strength of mortar.

The compressive and flexural strengths of mortar samples with different CSMP dosages are shown in Figure 6. In the first 28 days, mortars containing CSMP exhibit lower compressive and flexural strengths compared to the control group (0% CSMP). This reduction in strength becomes more pronounced as the CSMP dosage increases. At 28 days, the mortar with 10%, 20%, and 30% CSMP content showed a decrease of 0.9, 3, and 8.5 MPa in compressive strength, respectively, compared to the control group. At 54 and 90 days, the mortar with 10% CSMP exhibited a slight increase in compressive strength compared to the control group, while the compressive strength of the mortar with 20% and 30% CSMP content remained lower than that of the control group. This indicates that CSMP has pozzolanic activity and the optimal substitution ratio is 10%.

**Figure 7:**
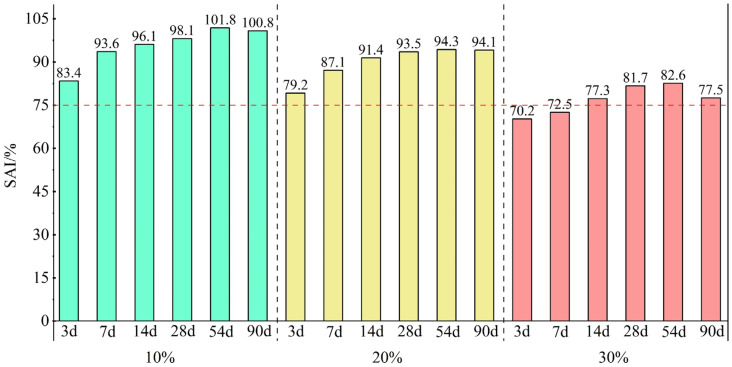
SAI of mortars with different CSMP dosages and at different curing ages.

Figure 7 shows the SAI of mortars with different CSMP dosages and at different curing ages. Mortar samples with 10% and 20% substitution levels meet the requirement of SAI > 75% specified in ASTM C618 [32] throughout all curing ages. After 14 days, mortar samples with a 30% substitution level also meet the specified requirements. Furthermore, at 54 and 90 days, the SAI of samples with a 10% substitution level exceeds that of the control group. Therefore, the optimal substitution rate for CSMP is 10%, and the 20% replacement rate can be applied to constructions with lower strength requirements.

**Figure 8:**
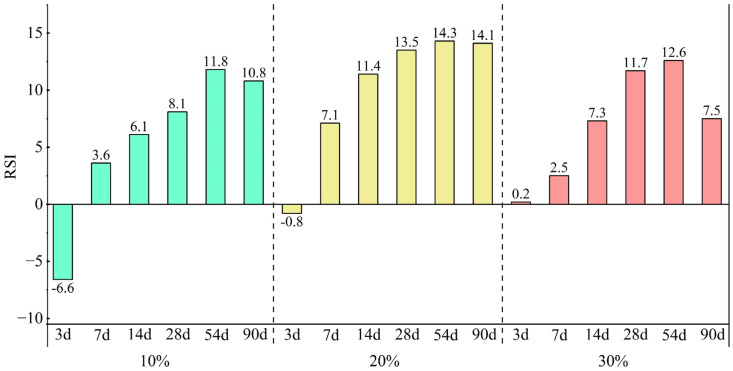
RSI of mortar at different CSMP dosages and at different curing ages.

Figure 8 presents the RSI of mortars with varying CSMP dosages and curing periods. At 3 days, the RSI values of samples with 10% and 20% substitution levels are negative but turn positive from the 7-day mark. For the 30% substitution level, the RSI remains positive throughout all curing ages. All mortar samples reach their maximum RSI at 54 days. This indicates that CSMP exhibits low pozzolanic activity during the early hydration stage, with its pozzolanic effect intensifying over time. This trend is consistent with the SAI results, suggesting that the optimal substitution rate for CSMP is 10%.

The above experimental results indicate that CSMP has potential as a supplementary cementitious material. However, compared to traditional materials, such as slag and metakaolin, its pozzolanic activity is limited. At higher substitution levels, CSMP significantly reduces the compressive and flexural strength of mortar, limiting its feasibility for applications requiring high mechanical performance. The strong water adsorption properties of clay minerals in CSMP adversely affect the workability of the mixture, with higher dosages resulting in substantially reduced fluidity [40]. Given its impact on both mechanical properties and workability, CSMP is not recommended as a supplementary cementitious material for concrete applications.

### 3.2. Performance Test Results of Synchronous Grouting Materials

#### 3.2.1. Fluidity and Consistency

**Figure 9:**
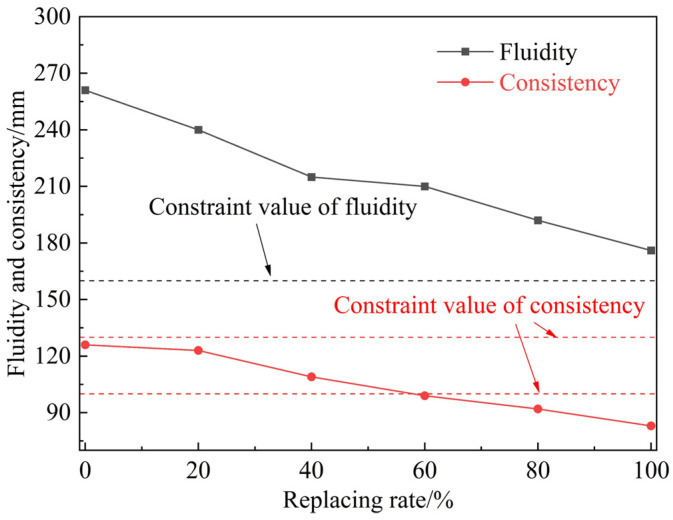
Relation curve of fluidity with replacement of fly ash.

The variation of fluidity and consistency of grouting with the fly ash replacement rate is shown in Figure 9. Due to the stronger water adsorption capacity of CSMP than fly ash, substituting fly ash with CSMP reduces the fluidity of the grouting, leading to a decrease in the pumpability of the material, with a more pronounced decline as the substitution rate increases. Although the fluidity of the grouting material remains within the specified range (>160 mm) according to the standard, when the fly ash substitution ratio exceeds 60%, the consistency falls below the minimum requirement (100 mm). At this point, the grouting material exhibits reduced fluidity and consistency, requiring higher injection pressures, which are unfavorable for pumping applications. Therefore, to ensure good pumpability and meet workability requirements, the substitution rate of CSMP for fly ash should not exceed 60%.

#### 3.2.2. Bleeding Rate and Hardening Rate

**Figure 10:**
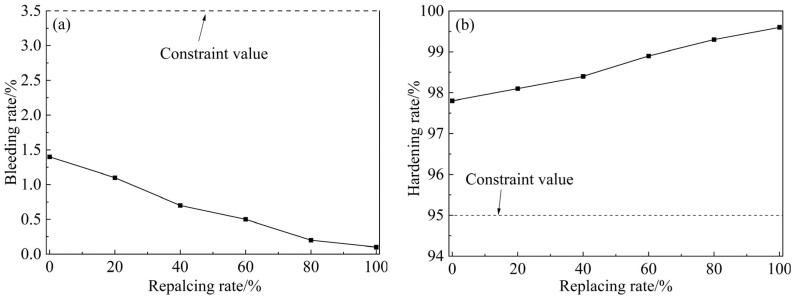
Relation curve of volume stability with replacement of fly ash: (**a**) Bleeding rate; and (**b**) Hardening rate.

Figure 10 shows the variation of the bleeding rate and hardening rate of grouting with the replacement rate of fly ash. As the fly ash replacement rate increases, the bleeding rate of the grouting decreases significantly. When CSMP completely replaces fly ash, the bleeding rate approaches zero, which is significantly lower than the standard in the specifications, which state the bleeding rate should be below 3.5%. This is attributed to the high specific surface area and charged layered structure of the clay minerals in CSMP, which provide strong water adsorption capacity, effectively fixing free water in the grouting and reducing the bleeding volume over time. The hardening rate of grouting remained above 95%, meeting the range specified by the regulations. Furthermore, the hardening rate increased with the fly ash replacement ratio. Thus, replacing fly ash with CSMP significantly enhances the volumetric stability of grouting and reduces ground loss caused by volume shrinkage, with a more pronounced effect at higher replacement rates.

#### 3.2.3. Setting Time and Compressive Strength

**Figure 11:**
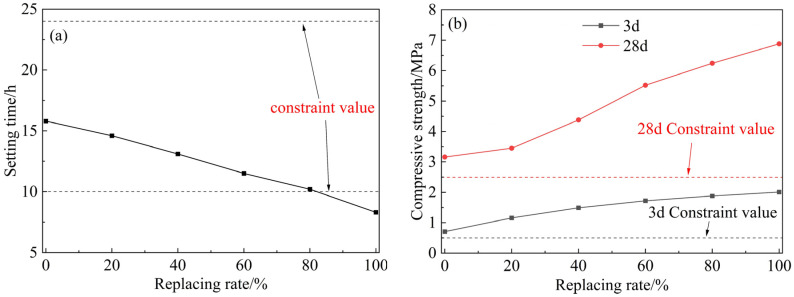
Relation curve of: (**a**) setting time; and (**b**) compressive strength with replacement of fly ash.

The variation of setting time and compressive strength of grouting with the fly ash replacement rate is shown in Figure 11. As the content of CSMP increases, the setting time significantly decreases. This is attributed to the stronger water adsorption capacity of CSMP compared to fly ash. Replacing fly ash with CSMP reduces the free water content in the grouting, effectively lowering the water-to-binder ratio. This decrease in free water reduces the dispersion of fine particles and enhances their cohesion, which results in a shorter setting time for the grouting. Additionally, the compressive strengths at 3 and 28 days increase with higher fly ash replacement rates, indicating that CSMP exhibits greater pozzolanic activity than the Class II fly ash used in the experiments.

CSMP can significantly improve the mechanical properties of the grouting and shorten its setting time. However, the setting time of the grouting material falls below 10 h, when the substitution rate of fly ash exceeds 80%, falling outside the standard-specified range of 10–24 h. Therefore, to reduce the construction difficulty of the synchronous grouting process and prevent pipeline blockage, based on the requirement for setting time, the substitution rate of fly ash with CSMP should not exceed 80%.

In summary, using CSMP to partially replace fly ash results in improved compressive strength, reduced bleeding, and enhanced stability of synchronous grouting materials. These changes contribute to better volumetric stability and reduced ground loss due to volume shrinkage. However, as the substitution ratio increases, the fluidity and consistency of the grouting decreases, and the setting time shortens, which can reduce the pumpability of the grouting material. A positive outcome of this is that it also enhances the ability of the material to constrain tunnel segments and prevent floating. Considering these factors, when the replacement rate is below 60%, the performance of the synchronous grouting material meets the standard requirements, demonstrating the feasibility of using CSMP in synchronous grouting applications.

### 3.3. Microstructural Analysis

#### 3.3.1. XRD

**Figure 12:**
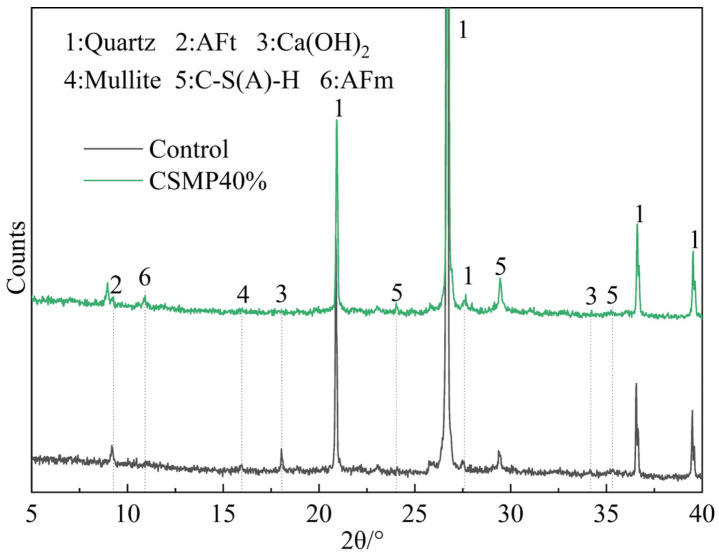
XRD patterns of 28 d Control and CSMP40% samples.

Figure 12 presents that XRD patterns of samples cured for 28 days with 0% replacement (Control) and 40% replacement (CSMP40%) reveal the presence of key hydration products, including Ca(OH)_2_, AFt (ettringite), AFm (monosulfide-type hydrated calcium thioaluminate), and C-S-H (calcium silicate hydrate), alongside mineral phases like quartz, feldspar, and mullite from the raw materials. The control sample shows pronounced diffraction peaks for Ca(OH)_2_ and C-S-H; whereas, the Ca(OH)_2_ peak in the CSMP40% sample almost disappears. In addition, compared to the control sample the C-S-H diffraction peak intensity is higher in CSMP40%. This indicates that CSMP exhibits higher pozzolanic activity than the fly ash used in the experiment, with its active Al_2_O_3_ and SiO_2_ reacting with the cement hydration product Ca(OH)_2_, consuming more Ca(OH)_2_, and generating additional gel substances. These gel substances can fill the voids in the grouting and effectively bond the cement particles and aggregates, enhancing the mechanical properties of the grouting [41,42].

In the control sample, the diffraction peaks for AFt are prominent, while the AFm peaks are relatively weak. In contrast, the CSMP40% sample exhibits stronger AFm peaks and weaker ettringite peaks. This indicates that CSMP promotes the transformation of AFt into AFm, likely due to the adsorption characteristics of CSMP. These characteristics reduce the content of free water in the grouting, accelerating the consumption of gypsum [43].

#### 3.3.2. SEM

**Figure 13:**
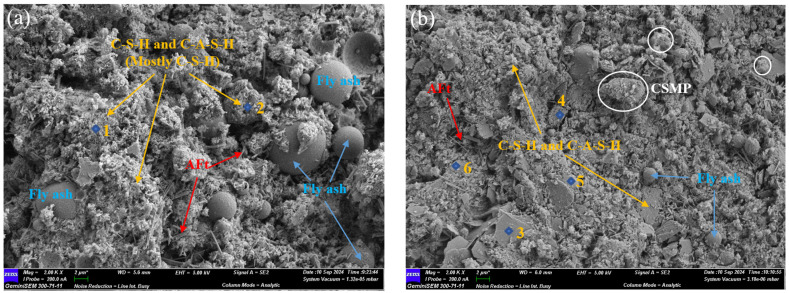
Microscopic morphology of 28 d samples of (**a**) Control and (**b**) CSMP40%.

Figure 13 presents SEM images of control and CSMP40% samples after 28 days of curing, while Table 4 presents the elemental content of each point in the SEM images. The Ca/Si for spectrums 1, 2, 4, and 5 are 2.38, 1.50, 1.69, and 1.07, respectively, with some aluminum content also being detected. These can be identified as mixtures of C-S-H and C-A-S-H. Both samples exhibit needle-like AFt crystals and unhydrated fly ash microspheres. In the control sample, the AFt phase is more widespread and evenly distributed, accompanied by a greater amount of unhydrated fly ash microspheres. In contrast, the CSMP40% sample contains more C-S-H or C-A-S-H, which results in a denser internal structure. Notably, neither sample shows obvious Ca(OH)_2_ crystals, which may be attributed to the low cement content in the grouting material and the consumption of Ca(OH)_2_ through volcanic ash reactions with the active SiO_2_ and Al_2_O_3_ present in the fly ash or CSMP.

Based on the elemental distribution in Table 4, compared to the control sample the C-A-S-H gel in the CSMP40% sample contains a higher aluminum content. This is due to the higher reactive Al_2_O_3_ content in the CSMP compared to the fly ash used in the experiment. The increased aluminum content in the CSMP40% sample enhances the complexity and order of the network structure in the C-A-S-H gel, stabilizing interlayer ions and promoting the formation of a dense layered structure. This reduces the porosity of the grouting material, thereby improving its compressive strength and durability [44,45]. The EDS results for spectrums 3 and 6 indicate the presence of significant amounts of Al and Si, along with small amounts of Ca, Mg, Fe, and K. These can be identified as unreacted clay particles and aluminosilicate mixtures.

#### 3.3.3. MIP

**Figure 14:**
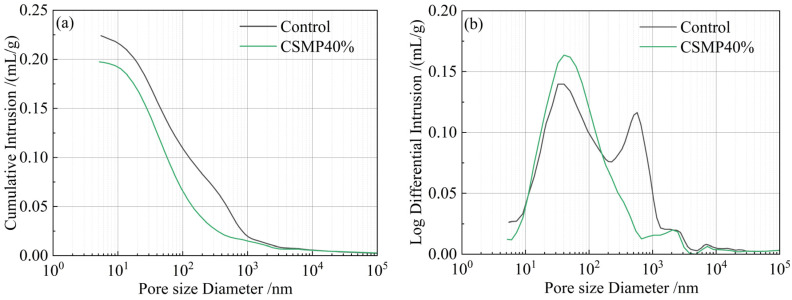
Distribution of pore size of 28 d samples of (**a**) control and (**b**) CSMP40% by MIP technology.

**Figure 15:**
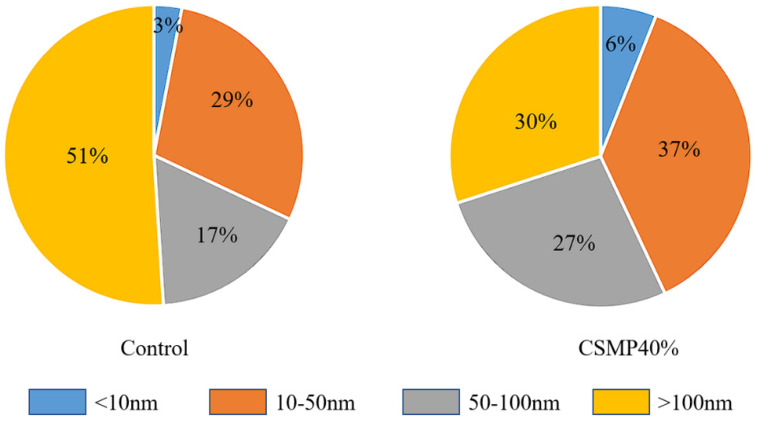
Proportion of different types of pores of 28 d samples of Control and CSMP40% observed by MIP technology.

Figure 14 shows the pore size distribution (PSD) and cumulative pore volume curves of the control sample and CSMP40% sample after 28 days of curing, obtained through MIP technology. The total porosity of the CSMP40% sample is significantly lower compared to the control sample. This is due to the higher pozzolanic activity of CSMP, which promotes the generation of gel substances, leading to a more compact internal structure of the grouting material. The pores in the grouting material are categorized into four types: (1) gel pores (pore size below 10 nm), (2) mesopores (pore size from 10–50 nm), (3) capillary pores (pore size from 50–100 nm), and (4) macro-pores (pore size > 100 nm). The distribution of these pore types for the control and CSMP40% samples is shown in Figure 15. Figure 15 illustrates that, in the control sample, mesopores and macro-pores dominate, making up 29% and 51% of the total pores, respectively. In contrast, the CSMP40% sample shows a more uniform pore size distribution, with 37% mesopores, 27% capillary pores, and 30% macro-pores. In the CSMP40% sample, the content of macro-pores larger than 100 nm is relatively lower, indicating an optimized pore structure. This difference can be explained by the excellent water-retention capacity of CSMP, which enables a more uniform distribution of the matrix within the grouting material. When water retention is poor, the tendency for larger droplets increases, which, in turn, results in more macro-pores [46,47]. The MIP results align well with the findings from XRD, SEM, and compressive strength tests, all of which indicate that CSMP enhances the compactness and overall performance of synchronous grouting materials.

## 4. Conclusions

Through the study of the pozzolanic activity of calcined shield muck powder and its re-utilization in synchronous grouting materials yielded the following key findings:

(1) Calcined shield muck powder shows some potential as a supplementary cementitious material. However, compared to traditional supplementary cementitious materials, its pozzolanic activity is limited. When the replacement dosage exceeds certain levels, it significantly reduces the mechanical properties and workability of the material. Therefore, calcined shield muck powder is unsuitable as a supplementary cementitious material for concrete applications.

(2) Compared to conventional synchronous grouting materials, replacing fly ash with calcined shield muck powder can enhance compressive strength, reduce bleeding rate, and increase hardening rate, all of which contribute to improved volumetric stability of the grouting material. However, fluidity and the consistency of grouting decreases, and setting time shortens, which may reduce pumping efficiency. Despite this, the shortened setting time helps to strengthen the restraint on tunnel segments and prevent segment uplift.

(3) Compared to the fly ash used in the experiment, calcined shield muck powder exhibits superior early pozzolanic activity, promoting the formation of C-S-H and C-A-S-H phases in grouting materials. This contributes to enhanced mechanical properties, optimized pore structure, and improved compactness and overall performance of the synchronous grouting materials.

(4) When the fly ash replacement rate is below 60%, the performance of grouting materials meet the standards and regulatory requirements, indicating the high feasibility of using CSMP as an alternative to fly ash in synchronous grouting materials.

## Figures and Tables

**Table 1:** Basic properties of white cement.

Density (g/cm^3^)	Specific Area (m^2^/kg)	Compressive Strength (MPa)		Flexural Strength (MPa)	
		3 d	28 d	3 d	28 d
3.15	340	29.4	50.3	5.9	8.6

**Table 2:** Chemical compositions of raw materials (%) obtained using X-ray fluorescence (XRF).

Materials	SiO_2_	Al_2_O_3_	CaO	Fe_2_O_3_	K_2_O	MgO	Na_2_O	TiO_2_	P_2_O_5_	SO_3_	Others
Cement	23.08	6.46	61.62	2.94	0.91	1.77	-	0.27	-	2.44	0.51
Shield Mud	65.80	28.45	1.05	1.51	1.55	0.57	0.26	0.37	-	-	0.44
Fly Ash	44.31	20.68	15.31	9.16	1.21	1.99	3,81	-	0.32	1.59	1.7
Bentonite	67.57	18.26	2.33	3.82	2.66	1.95	2.59	0.47	0.15	0.08	0.12

**Table 3:** Performance requirements of synchronous grouting and measured values of reference group.

Performance	Fluidity (mm)	Consistency (mm)	Setting Time (h)	Bleeding Rate (%)	Hardening Rate (%)	Compressive Strength (MPa)	
						3 d	28 d
Measured value	261	126	15.8	1.4	97.8	0.71	3.16
Standard limit [29]	>160	100–130	10–24	<3.5	>95.0	>0.50	>2.50

**Table 4:** The elemental content of each point in SEM-EDS (%).

Spectrum	O	Mg	Al	Si	Ca	Na	Fe	K
1	49.05	2.23	3.81	8.56	20.42	0.51	3.46	0.22
2	49.86	1.11	3.90	11.83	17.75	0.64	1.90	0.35
3	53.88	0.75	12.24	12.48	4.68	-	1.28	2.17
4	53.18	0.97	8.09	8.03	13.58	-	1.33	0.37
5	55.00	2.16	7.52	9.77	10.53	-	1.65	0.33
6	49.45	4.51	8.06	13.09	4.27	-	4.76	2.58

## Data Availability

The original contributions presented in this study are included in the article. Further inquiries can be directed to the corresponding author.
